# On the Three Common Intestinal Entamoebae of Man, and Their Differential Diagnosis

**Published:** 1918-05

**Authors:** 


***On the Three Common Intestinal Entamoebae of Man, and their Differential Diagnosis.*** By CLIFFORD DOBELL, M. A., and MARGARET W. JEPPS. (Report to the Medical Research Committee). *Brit. Med. Journ.*, May 12, 1917.

The authors describe their observations of the *Entamoeba nana* of Wenyon and O'Connor, and the methods they have found most useful in identifying this organism and differentiating it from the *E. histolytica*, the *E. coli* and other organisms in the routine examination of human faeces for the detection of protozoal infections.

*Entamoeba nana.* — The amoeba is very small, measuring, when rounded, about 6 to 12 μ in diameter, the majority being about 8 μ. In general structure it somewhat resembles many amoebae of the so-called *limax* group. The protoplasm is generally much vacuolated, the vacuoles containing bacteria upon which the animal feeds. There is no contractile vacuole. The pseudopodia are few, blunt, and hyaline. The very characteristic nucleus is rather small, and not always observable in the living organisms. Its karyosome is smaller than that in the nucleus of a typical A. *limax*, and usually occupies an apparently excentric or peripheral position. It is usually irregular in shape and often segmented into several pieces of unequal size united by slender threads. The amoeba moves very sluggishly on a cold stage, and very soon becomes rounded up and dies. Attempts at cultivation on amoeba sugar have always failed.

The cysts are oval or spherical structures, usually measuring 8 to 10 μ in length or 7 to 8 μ in diameter. The nuclei are 1, 2, or 4 in number in the vast majority of cysts, closely resembling in structure those of the unencysted amoebae except for their smaller size in late stages of development. In the quadrinucleate cyst the nuclei usually lie near to one another and frequently in close contact. Very rarely cysts have been observed containing as many as eight nuclei. In addition to the nuclei the cysts contain a variable number of highly refringent granules which give some of the reactions of volutin; and sometimes — especially in the binucleate stage — a large dull inclusion is also visible in the living cyst. This is composed of a substance insoluble in alcohol, soluble in water, colored dark brown by iodine, and bright red with Best's carmine, from which it would appear to be a mass of glycogen. The cysts seem to be very resistant; they cannot withstand drying, but remain apparently unchanged in moist faeces for more than three weeks. From the fact that persons infected with *E. nana* sometimes pass diarrheic stools which contain only cysts, it seems not improbable that the habitat of this amoeba is the small intestine. The *E. nana* is a very common parasite, has a wide geographical distribution, and there is no evidence to show that it is pathogenic.

*Entamoeba histolytica.* Examination of 200 specimens showed the cysts to be of very varying sizes. In 75, they were below 10 μ in diameter, generally between 7 to 9 μ; in 127, they were between 10 and 15 μ, the majority ranging around 12; and in 4, cysts as large as 16 μ, 16.5 μ, and 17.6 μ were recorded.

*Entamoeba coli.* The authors do not agree with other writers that the cysts of *E. coli* can be distinguished from those of *E. histolytica* by their size. They consider the only certain means of differentiating them is by the study of the structure and number of the nuclei. They themselves have never found those of the *E. histolytica* containing more than four; and therefore regard the occurrence of cysts containing more than four nuclei of the *coli-histolytica*

type as the most certain criterion of an *E. coli* infection. In this they agree with Mathis and Mercier and differ from Swellengrebel and Schiess.

The authors emphasize the fact that, in the absence of unencysted forms, specific diagnosis of an entamebic infection is frequently very difficult and not seldom impossible. From the encysted form, however, a correct diagnosis can usually be made with certainty by a practised observer.

The presence of chromatoid bodies, generally present in *E. histolytica* cysts, are, contrary to the statements of Mathis and Mercier, not uncommon in *E. coli* cysts. A much more distinctive character is the vacuole, which is formed in the early stages of encystation and persists as a rule in *E. histolytica* until the 4-nucleate stage is reached. In *E. coli* it is seen up to the 4-nucleate stage, but is very uncommon in cysts containing 8 nuclei. It attains its maximum size in *E. coli* during the binucleate stage, when it frequently occupies the greater part of the cyst. Unlike those of *E. coli*, *E. histolytica* cysts often possess more than one vacuole. The vacuoles are best studied in cysts mounted in iodine solution. As those of *E. coli* contain abundant glycogen, they stain dark brown; whereas *E. histolytica* vacuoles, which contain but little of this substance, are far less deeply colored. In iodine solution the edge of the vacuole is ill-defined in *E. histolytica*, but usually clear cut in *E. coli*; but these characters are not absolutely constant, for cysts of *E. histolytica* with vacuoles quite as darkly stained and sharply outlined as those of *E. coli* have been seen. The thickness of the cyst wall, regarded by some writers as a reliable character for distinguishing the cysts of *E. coli* from those of *E. histolytica*, seems to have no importance in the differentiation.

The cysts of *E. nana* are readily distinguished from those of *E. histolytica* by the structure of their nuclei, the absence of chromatoid bodies, and the character of the glycogen vacuoles. Moreover, although great variation is observable in the shape of the cysts of both species, those of *E. nana* are generally oval, while those of *E. histolytica* are typically spherical. The nuclei of small histolytica cysts are seldom distinctly visible when the cysts are examined in saline solution; while those of *E. nana* cysts are, for all practical purposes, completely invisible in this medium. In iodine solution the nuclei of *E. histolytica* cysts can generally be clearly made out, while this is seldom the case with cysts of *E. nana* on account of the numerous minute granules (? volutin) which are generally present. As a rule the chromatoid bodies of *E. histolytica* cysts are readily seen in saline or iodine (especially in the former), and when present serve at once to distinguish them. The glycogen vacuoles should be studied in iodine solution, in which the small, faintly stained, and ill-defined vacuole of *E. histolytica* is easily distinguished from the relatively large, deeply stained, and sharply defined glycogen mass of *E. nana*.

The cysts of *E. histolytica* may also be confused with those of *Chilomastix* (= *Tetramitus*), which are often present in human faeces. Although these cysts are sometimes oval and even spherical, by far the commonest form possesses a protuberance at one end, so that in shape it closely resembles a lemon. They measure about 7 to 10 μ in greatest diameter. They never contain more than one nucleus, which is of large size and characteristic in structure. As the chromatin is usually situated at one pole, it is typically in the form of a signet ring. Smaller masses of chromatin are sometimes visible at other points on the nuclear membrane but rarely in the centre. For this reason the nuclei of encysted *Chilomastix* often resemble those of the uninucleate cysts of *E. nana*. This resemblance is most striking in stained preparations; for in cysts examined in saline the nuclei of both are usually invisible, whilst in iodine, although the *Chilomastix* nucleus can usually be made out as a definite signet ring only the karyosome of the nucleus of *E. nana* can be distinguished with ease. The most distinctive feature, however, of the *Chilomastix* cyst is a complex structure which represents the persistent part of the mouth of the free flagellate. This structure resembles a sling, its outline representing the fiber forming the lip of the mouth. The mouth flagellum, which in the flagellate borders an undulating membrane, can sometimes be seen lying within the sling. The relation of these parts to the nucleus and blepharoblasts is similar to that seen in the free flagellate, but in the encysted forms the connexion between these structures is often severed. The outline of the mouth, though usually invisible in cysts examined in saline, can as a rule be clearly seen in iodine preparations. Another feature, characteristic of the *Chilomastix* cyst is the presence of a few small and brightly refractile granules which appear to be composed of volutin. The similar granules in the cysts of *E. nana* are usually smaller, less refractile, and more numerous. Another character, which does not appear to have been previously noted, is the occasional presence in the *Chilomastix* cyst of a mass of glycogen. This mass, which stains very deeply in iodine solution, is sometimes of very large size and seems to be present in newly-formed cysts only. The oval cysts of *Chilomastix* containing glycogen may be mistaken for similar cysts of *E. nana*; and both must be distinguished from the usually much larger cyst-like bodies described as " I-cysts " by Wenyon (1916) and Wenyon and O'Connor (1917), which are not uncommon in human faeces.

From the foregoing descriptions it will be evident that, when the cysts are examined in saline and iodine solutions, the most outstanding characters differentiating those of *E. histolytica*, *E. nana*, and *Chilomastix* from one another are : the chromatoid bodies of the first, the apparently uniformly granular contents of the second, and the mouth of the third.

